# Early Prognostic Indicators of Subsequent Hospitalization in Patients with Mild COVID-19

**DOI:** 10.3390/jcm10081562

**Published:** 2021-04-08

**Authors:** Alyssa Ylescupidez, Aaron Rips, Henry T. Bahnson, Cate Speake, Punam Verma, Anne M. Hocking, Jane H. Buckner, Uma Malhotra

**Affiliations:** 1 Center for Interventional Immunology, Benaroya Research Institute at Virginia Mason, Seattle, WA 98101, USA; AYlescupidez@benaroyaresearch.org (A.Y.); arips@student.nymc.edu (A.R.); TBahnson@benaroyaresearch.org (H.T.B.); CSpeake@benaroyaresearch.org (C.S.); 2 Department of Microbiology, Virginia Mason Medical Center, Seattle, WA 98101, USA; punam.verma@vmmc.org; 3 Center for Translational Immunology, Benaroya Research Institute at Virginia Mason, Seattle, WA 98101, USA; AHocking@benaroyaresearch.org (A.M.H.); jbuckner@benaroyaresearch.org (J.H.B.); 4 Department of Infectious Disease, Virginia Mason Medical Center, Seattle, WA 98101, USA; 5 Department of Medicine, Section of Infectious Diseases, University of Washington, Seattle, WA 98101, USA

**Keywords:** COVID-19, SARS CoV-2, pathophysiology, prognostic indicators, pandemic, comorbidities, lymphopenia, shortness of breath

## Abstract

Comprehensive data on early prognostic indicators in patients with mild COVID-19 remains sparse. In this single center case series, we characterized the initial clinical presentation in 180 patients with mild COVID-19 and defined the earliest predictors of subsequent deterioration and need for hospitalization. Three broad patient phenotypes and four symptom clusters were characterized, differentiated by varying risk for adverse outcomes. Among 14 symptoms assessed, subjective shortness of breath (SOB) most strongly associated with adverse outcomes (odds ratio (OR) 21.3, 95% confidence interval (CI): 2.7–166.4; *p* < 0.0001). In combination, SOB and number of comorbidities were highly predictive of subsequent hospitalization (area under the curve (AUC) 92%). Additionally, initial lymphopenia (OR 21.0, 95% CI: 2.1–210.1; *p* = 0.002) and male sex (OR 3.5, 95% CI: 0.9–13.0; *p* = 0.05) were associated with increased risk of poor outcomes. Patients with known comorbidities, especially multiple, and those presenting with subjective SOB or lymphopenia should receive close monitoring and consideration for preemptive treatment, even when presenting with mild symptoms.

## 1. Introduction

Most studies of COVID-19 have focused on hospitalized patients with severe and critical disease [1,2,3,4]. Comprehensive data on the clinical picture and early prognostic indicators in ambulatory patients with COVID-19 remains limited [5,6,7]. With a novel disease, such data are critical to our understanding of disease prognosis and recovery in those presenting with mild illness, while also selecting appropriate candidates for emerging preemptive treatment strategies. Many patients with COVID-19 present with a classic biphasic illness, first with mild symptoms and then with a second phase consisting of hypoxia and respiratory distress. In this study, we characterized the clinical presentation of patients presenting with mild illness to discern the earliest predictors of subsequent deterioration and need for hospitalization. Such findings are important for allocating resources, providing informed patient counseling, and knowing when to be especially alert for complications.

## 2. Materials and Methods

### 2.1. Study Population

This study of 180 consecutive adult patients with COVID-19 seen in the ambulatory setting at the Virginia Mason Medical Center, Seattle, Washington, USA, was approved by the Benaroya Research Institute at Virginia Mason’s Institutional Review Board (IRB20-075). All patients were confirmed COVID-19 positive by reverse transcriptase-polymerase chain reaction (RT-PCR) for SARS-CoV-2 on nasopharyngeal swabs. Patients were discharged home based on clinician judgment. Electronic medical records were reviewed to collect clinical and demographic information on a standardized collection form. Comorbidities were assessed through review of patient medical history and current BMI.

### 2.2. Statistical Analyses

Patient outcome was assessed as a binary outcome, home recovery or subsequent hospitalization, and as an ordinal outcome, further distinguishing home recovery patients between those with and without complications. Two-way hierarchical clustering analysis, including all symptoms, was used to identify patient clusters and variable clustering identified clusters of co-occurring symptoms. Recursive partitioning was used to determine the optimal decision tree between presenting symptoms and the binary patient outcome (home recovery or subsequently hospitalized). Ordinal logistic regression was implemented to identify predictors of outcome severity ordered by hospitalized, returned with worsening or new symptoms but not hospitalized, and home recovery with no further complications after initial presentation. To account for small sample size and infrequency of subsequent hospitalization, Firth bias-adjusted generalized linear models were employed. Associations between variables were assessed by Pearson’s correlation coefficient. For group comparisons of continuous data, Kruskal–Wallis tests or ANOVA were used where appropriate; chi-square or Fisher’s exact two-sided tests were used for categorical data. All tests were at the alpha = 0.05 significance level. Analyses were performed using SAS 9.4 (SAS Institute Inc., Cary, NC, USA) and JMP Pro 15 (SAS Institute Inc., Cary, NC, USA).

## 3. Results

### 3.1. Demographic, Clinical and Laboratory Characteristics and Outcome

**Figure 1:**
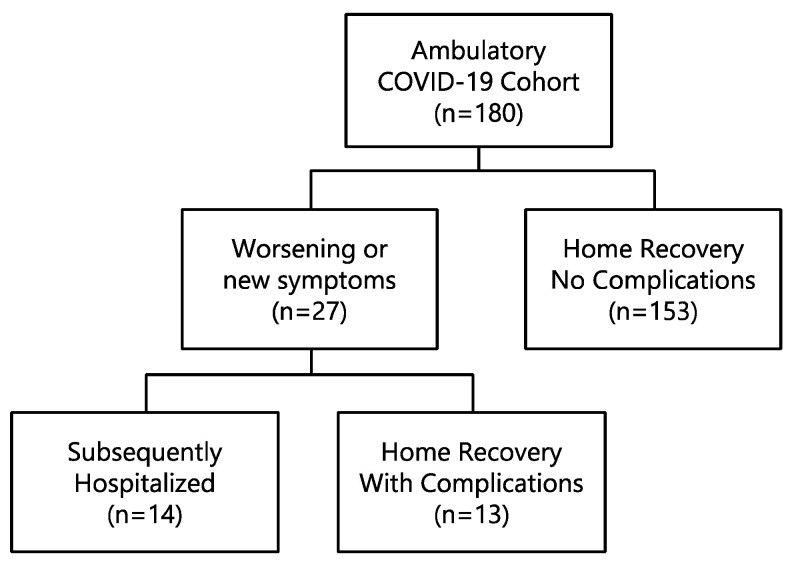
Patient groups based on clinical outcome. Of the 180 adult patients, 153 completed home recovery without complications (Home Recovery No Complications group), 27 experienced subsequent worsening or new symptoms after discharge with 14 of these requiring hospitalization (Subsequently Hospitalized group), and the remaining 13 completing home recovery (Home Recovery With Complications group). The Combined Home Recovery group consisted of both Home Recovery No Complications and Home Recovery With Complications (*n* = 166).

Between 10 March and 31 May 2020, 180 adult patients with COVID-19 were seen in the ambulatory setting at our medical center presenting a median of 5 days after symptom onset (range 1–60 days). Patients were then grouped based on whether they completed home recovery without complications (Home Recovery No Complications; *n* = 153), completed home recovery with complications (Home Recovery With Complications; *n* = 13), or developed complications requiring hospitalization (Subsequently Hospitalized; *n* = 14) (Figure 1). In the Home Recovery With Complications group, ten patients had pneumonia of varying severity, two were diagnosed with bronchitis, and one experienced atrial fibrillation requiring cardioversion. Further evaluation of one of these patients led to diagnosis of adenocarcinoma of the lung with subsequent surgical resection. Patients in the Subsequently Hospitalized group presented a median of five days (range 1–22 days) after initial presentation and included 13 patients with progressive respiratory failure with two patients succumbing to the illness, and one patient hospitalized for severe periorbital cellulitis.

To define predictors of subsequent clinical deterioration with development of complications or requiring hospitalization, we compared the demographics and initial clinical presentation of the three groups (Table 1). The Home Recovery No Complications group was younger (median age of 52.6 versus 56.5 and 56 years for the Home Recovery With Complications and the Subsequently Hospitalized groups, respectively (*p* = 0.05). Males were at greater risk for both subsequent complications and need for hospitalization (binary outcome, odds ratio (OR) 3.5, 95% confidence interval (CI): 0.9–13.0; *p* = 0.05; three group outcome through ordinal logistic regression, OR 2.9, 95% CI: 1.2–7.3; *p* = 0.02). In the Home Recovery No Complications group, 50.3% (77/153) were female, compared to 30.8% (4/13) of the Home Recovery With Complications and 21.4% (3/14) of the Subsequently Hospitalized. Race and ethnicity were similar across the groups as well as duration of symptoms at initial presentation.

**Figure 2:**
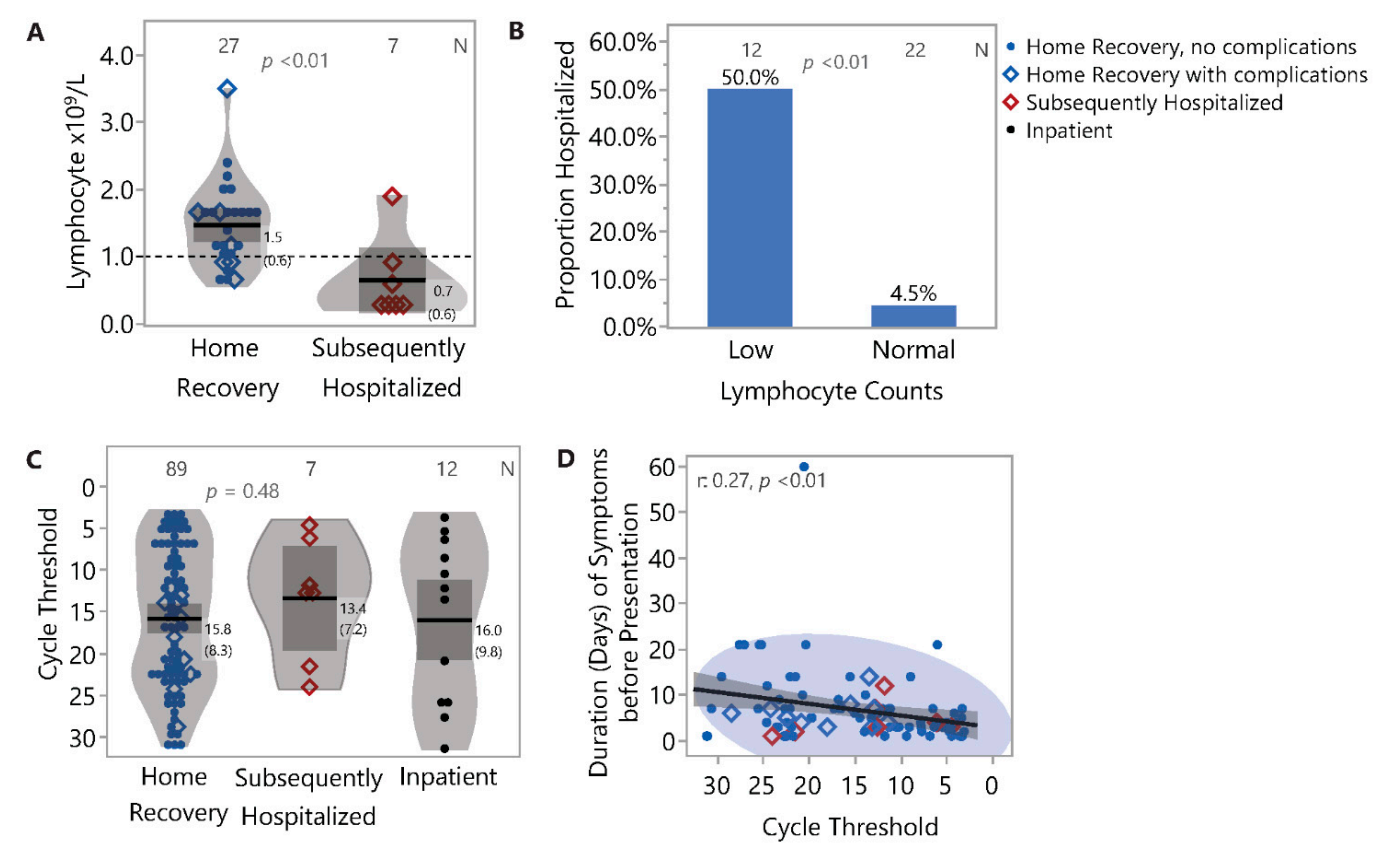
Association of lymphocyte counts and virus cycle threshold with clinical outcome. (**A**) Lymphocyte counts were lower in the Subsequently Hospitalized group compared to the Home Recovery group. Further, within the Home Recovery group, 50% (3/6) of those with lymphopenia had complications compared to 19% (4/21) among those with normal counts. (**B**) Low lymphocyte counts (<1.0 × 10^9^ cells/L) were associated with increased risk for subsequent hospitalization. Fifty percent (6/12) of participants with low lymphocyte counts were subsequently hospitalized compared to only 4.5% (1/22) of participants with normal counts. (**C**) Cycle threshold data, a surrogate for viral load, was not significantly associated with outcomes. Lower cycle threshold values indicate higher levels of viral nucleic acid. (**D**) Duration of illness at presentation was inversely associated with the level of virus.

Only 34 of 180 patients had blood work including a Complete Blood Count (CBC) at initial presentation. Of these 34 patients, 12 had lymphopenia (<1.0 × 10^9^ cells/L) which was associated with overall poor outcomes, including increased risk for complications during home recovery as well as need for subsequent hospitalization (OR 21.0, 95% CI: 2.1–210.1; *p* = 0.002). Lymphocyte counts were lower in the Subsequently Hospitalized group compared to the Combined Home Recovery group (ANOVA *p* = 0.004) (Figure 2A). Conversely, patients with lymphopenia had a high likelihood of requiring subsequent hospitalization (50%, 6/12) versus those without lymphopenia (4.5%, 1/22) (Figure 2B). Moreover, all 4 patients with lymphocyte counts <0.5 × 10^9^/L required hospitalization.

We also investigated if viral load in the nasopharyngeal swab was predictive of outcome using RT-PCR cycle threshold (Ct) as a surrogate for the quantity of virus RNA (Figure 2C). We limited this analysis to the 108 patients in our cohort that had been diagnosed using the Abbott *m*2000 platform in order to avoid confounders related to assay platform differences. There was no significant association between disease outcome and initial viral load in terms of risk for subsequent hospitalization or complications. We also did not observe a significant difference in the viral load between these patients versus an inpatient control group hospitalized upon initial presentation (*n* = 12). However, duration of illness at presentation was inversely associated with the level of virus (Pearson correlation coefficient = 0.27, *p* = 0.0098) (Figure 2D).

### 3.2. Symptomatology and Symptom Clusters 

We determined whether any of the 14 assessed symptoms were individually predictive of subsequent outcome (Table S1). Presence of subjective shortness of breath (SOB) was the strongest predictor of subsequent complications as well as hospitalization (binary OR 21.3, 95% CI: 2.7–166.4; *p* < 0.0001). SOB was reported in 35.9% of the Home Recovery No Complications group, 61.5% Home Recovery With Complications group, and 92.9% of the Subsequently Hospitalized group. Fatigue was also seen at a higher frequency among the Subsequently Hospitalized group versus the Combined Home Recovery group (42.9% versus 16.9%; binary OR 3.7, 95% CI: 1.2–11.5; *p* = 0.02). Cough, fever, chills, and myalgias were common but their presence or absence was not predictive of subsequent outcome. Diarrhea, sinus pressure, nasal congestion, headache, sore throat, chest pain, anorexia/nausea were observed at lesser frequencies, and also not predictive of subsequent outcome. Interestingly, only outpatients with uneventful recoveries reported loss of taste or smell (14.4% of Home Recovery No Complications). This difference could be related to a preponderance in involvement of the lower respiratory tract in the Subsequently Hospitalized group. 

**Figure 3:**
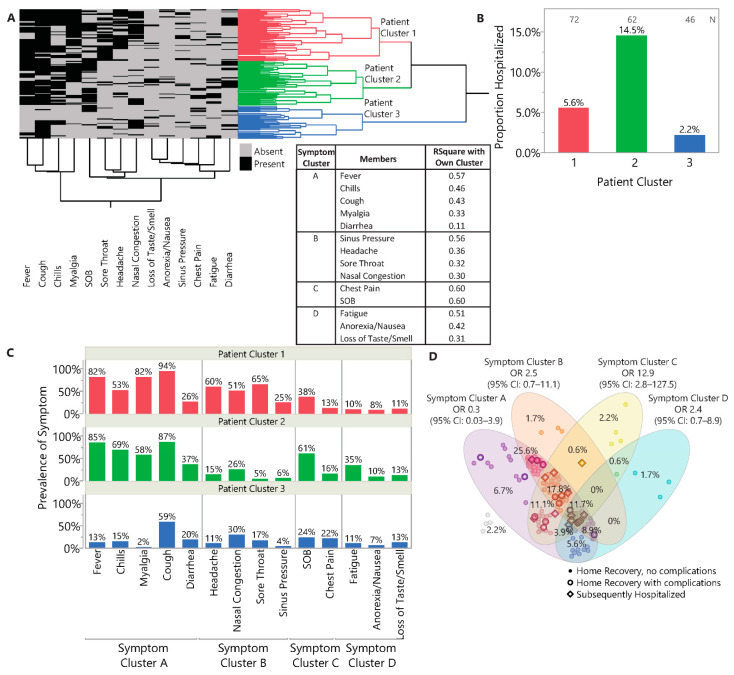
Symptom clusters using hierarchal and variable clustering. (**A**) 2-way hierarchical clustering of symptoms shows three patient clusters/phenotypes and four clusters of co-occurring symptoms: symptom cluster A: flu-like with fevers, chills, cough, myalgia, diarrhea; symptom cluster B: upper respiratory, with nasal congestion, sinus pressure, sore throat, and headache; symptom cluster C: pneumonia-like, with shortness of breath and chest pain; and symptom cluster D: anorexia/nausea, fatigue, loss of smell/taste. (**B**) Patient cluster 2 is most associated with hospitalization, while cluster 3 had the lowest hospitalization rate. (**C**) Overall, cluster 1 patients had high prevalence of symptom cluster A (flu-like) and B (upper respiratory) symptoms. Cluster 2 patients experienced high prevalence of symptom cluster A (flu-like) and C (pneumonia-like) symptoms, including the highest prevalence of SOB among all patient clusters. Cluster 3 patients experienced few symptoms with the exception of cough. (**D**) Association of symptom clusters with outcome. Thirteen of 14 admitted patients had symptoms from Cluster C. Each data point represents a single patient and the marker symbols indicate types of outcomes. Four outpatients were asymptomatic. Odds ratio with 95% CI for the association with subsequent hospitalization for each symptom cluster are annotated at the top of the Venn diagram.

Using two-way hierarchal clustering of symptoms, we observed three patient clusters/phenotypes (Figure 3A). Patient Cluster 2 was most associated with hospitalization (14.5%, 9/62), while Patient Cluster 3 had the lowest hospitalization rate (2.2%, 1/46) (Figure 3B). Symptom clustering determined which symptoms grouped together/co-occurred, resulting in four symptom clusters: A, *flu-like* with fevers, chills, cough, myalgias, and diarrhea; B, *upper respiratory*, with nasal congestion, sinus pressure, sore throat, and headache; C, *pneumonia-like*, with SOB and chest pain; and D, anorexia/nausea, fatigue, and loss of smell/taste. Patient Cluster 1 had high prevalence of flu-like and upper respiratory symptoms, Patient Cluster 2 had high prevalence of flu-like and pneumonia-like symptoms, including the highest prevalence of SOB among the three patient clusters. Patient Cluster 3 had few symptoms, with the exception of cough (Figure 3C).

Patients often presented with symptoms from multiple symptom clusters (154/180, 85.6%) (Figure 3D). The entire Subsequently Hospitalized group exhibited symptoms from multiple symptom clusters with 42.8% (6/14) showing symptoms from all four symptom clusters compared to only 9% (15/166) of the Home Recovery groups. We also assessed how symptom clusters associated with patient outcome in a multivariable model adjusting for age, BMI, and sex. Probability of subsequent hospitalization was highest when patients presented with pneumonia-like symptoms (OR 12.9, 95% CI: 2.8–127.5; *p* = 0.003), followed by upper respiratory symptoms (OR 2.5, 95% CI: 0.7–11.1; *p* = 0.19), and symptoms in Symptom Cluster D (anorexia/nausea, fatigue, loss of taste/smell; OR 2.4, 95% CI: 0.7–8.9; *p* = 0.18). Flu-like symptoms were associated with more favorable outcomes (OR 0.3, 95% CI: 0.03–3.9; *p* = 0.27). 

### 3.3. Comorbidities and Risk Factors

**Figure 4:**
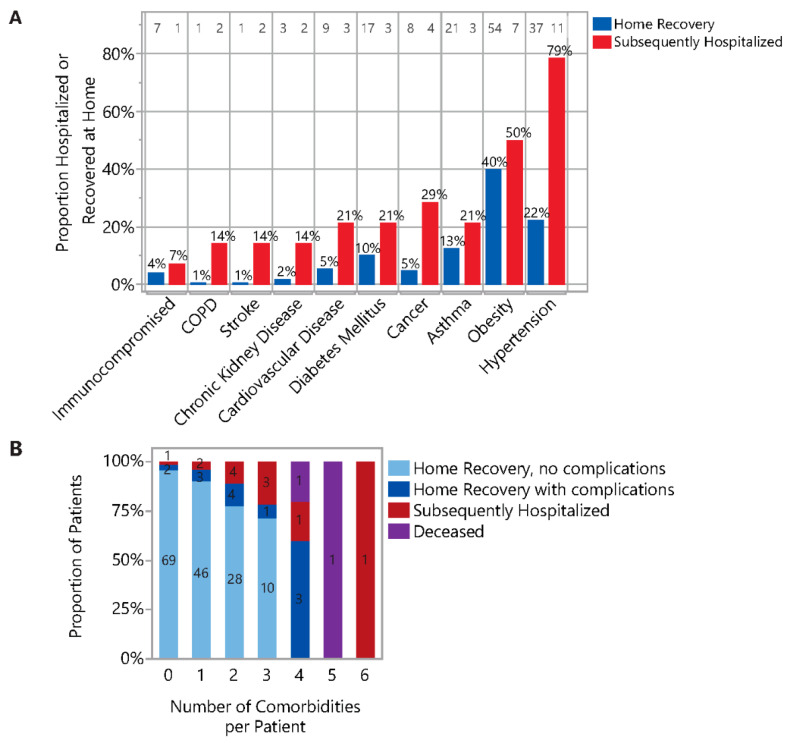
Comorbidities are of high prognostic value. (**A**) Proportion of patients with individual comorbidities in the Home Recovery and the Subsequently Hospitalized groups. Number of patients with indicated comorbidity are annotated along the top of the figure and top of bars are annotated with proportion of Home Recovery or Subsequently Hospitalized group. BMI was unknown for 31 Home Recovery patients. (**B**) Increasing numbers of risk factors corresponded with increasing risk of adverse outcome. Probability of home recovery declined steadily with increasing comorbidities.

In the Home Recovery No Complications group, 60.1% (92/153) had no known comorbidities, versus 23.1% (3/13) and 14.3% (2/14) in the Home Recovery With Complications group and the Subsequently Hospitalized, respectively (Table 1) (*p* = 0.0003). Fifty percent of the Subsequently Hospitalized and 40% of the Home Recovery groups were obese (BMI ≥ 30 kg/m^2^), although obesity alone was not a significant predictor of the binary or ordinal outcome (binary OR 1.5, 95% CI: 0.5–4.5; *p* = 0.47). Hypertension was another common comorbidity in both the Home Recovery and Subsequently Hospitalized (78.6% and 22.0%, respectively) (Figure 4A). Other comorbidities included asthma (12.7% Home Recovery versus 21.4% Subsequently Hospitalized), cancer (4.8% versus 28.6%), diabetes mellitus (10.2% versus 21.4%), cardiovascular disease (5.4% versus 21.4%), chronic kidney disease (1.8% versus 14.3%), prior stroke (0.6% versus 14.3%), COPD (0.6% versus 14.3%), and immunocompromised state (4.2% versus 7.1%). Of the admitted patients, 46% were current or former smokers compared with 26% of the Home Recovery (*p* = 0.11). 

The total number of risk factors (the cumulative number of known comorbidities and obesity) was highly associated with both the binary and ordinal patient outcomes (Figure 4B; *p* < 0.0001 for both), and admitted patients had more risk factors compared to outpatients (Subsequently Hospitalized: median 3, range 0–6 versus Combined Home Recovery: median 1, range 0–4, *p* < 0.0001). Of patients with no comorbidities, 96% had an uneventful home recovery. This proportion decreased to 90% (46/51) in those with a single comorbidity, 78% (28/36) in those with two, and 71% (10/14) in those with three. All seven patients with four or more comorbidities experienced complications, four of which (57%) required subsequent hospitalization, including two who succumbed to illness (29%). Notably, the total number of risk factors was identified to be of high prognostic value–logistic regression determined an area under the curve (AUC) of 82% when predicting the binary outcome. Furthermore, a multivariable model with SOB and number of risk factors as covariates increased AUC to 92%. 

### 3.4. Duration of Illness and Viral Load: Association with Symptoms

We did not observe a significant association between duration of illness before presentation and presence or absence of individual symptoms (Figure S1). We then assessed if viral load was associated with the presence or absence of symptoms (Figure S2). The presence of fever was statistically significantly associated with higher levels of virus (*p* = 0.01), while presence of chills and myalgias showed a trend towards association (*p* = 0.07). Other symptoms did not show a significant association with virus level.

### 3.5. Outcome Analyses

**Figure 5:**
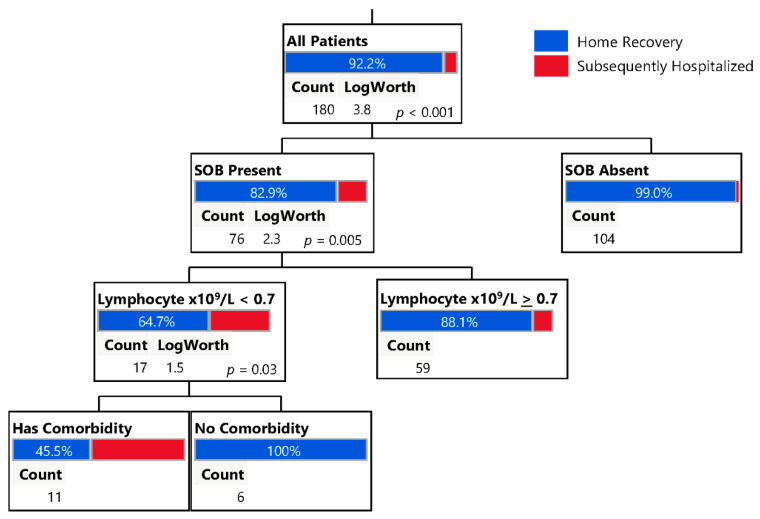
Outcome decision tree analyses. Partitioning of data, where patient outcome is a binary outcome (Home Recovery or Subsequently Hospitalized). Variables included in this analysis were all symptoms, presence of any known comorbidities, lymphocytes, cycle threshold, age, BMI, and sex. Partitioning identifies SOB (*p* < 0.001), lymphocyte counts (*p* = 0.005), and comorbidities (*p* = 0.03) to be most associated with need for hospitalization. Observations with missing data are randomly and iteratively assigned to one of two sides of the split to utilize the entire cohort.

To assess the impact of various parameters on outcome, we partitioned the data from the ambulatory cohort (*n* = 180), where patient outcome was a binary event (Combined Home Recovery or Subsequently Hospitalized) (Figure 5). Variables included in this analysis were all symptoms, presence of any known comorbidities, lymphocytes, cycle threshold, age, BMI, and sex. SOB (*p* = 0.0002), lymphocyte counts (*p* = 0.005), and presence of a comorbidity (*p* = 0.03) were most associated with patient outcome. Of patients who did not report SOB at initial presentation only 1% required subsequent hospitalization. By contrast, among those that reported SOB at initial presentation, 17% required subsequent hospitalization. The concurrent presence of lymphopenia (counts <0.7 × 10^9^/L) with SOB further increased the risk for subsequent hospitalization to 35%. Furthermore, the concurrent presence of a comorbidity, along with lymphopenia and SOB, increased the risk to 55%.

## 4. Discussion

To improve clinical outcomes among patients with COVID-19, evidence-based literature is essential to guide providers in the management of patients across the disease spectrum. Just as there is lack of consensus in the optimal treatment of hospitalized patients [8], there are knowledge gaps in the appropriate triage and management of ambulatory patients with mild disease. A more comprehensive understanding of the factors predictive of subsequent clinical deterioration allows for targeted intensive monitoring and treatment, which may help improve outcome and prognosis in this group. 

In this study, we characterized the initial clinical presentation of 180 adult patients with COVID-19 in the ambulatory setting to determine early prognostic indicators of subsequent hospitalization. Consistent with the literature [9], the presence of comorbidities was the strongest predictor of complications. We observed a striking association between the cumulative number of risk factors and adverse outcomes, with a steady increase in proportions of poor outcomes with increasing comorbidities. Although an association between comorbidities and COVID-19 mortality is well established, the number of comorbidities and presenting symptoms as a predictor of subsequent hospitalization among initially mild cases is less understood. Here we show that the cumulative number of risk factors alone had high prognostic value, with an AUC of 82% in prediction of subsequent hospitalization. Predictive ability was further strengthened when SOB was combined with cumulative risk factors (AUC 92%). Accurate prediction of adverse outcomes in this setting may enable clinicians to efficiently triage patients presenting with mild symptoms and prevent the worsening of disease. Further analysis of predictors of mortality was beyond the scope of this work given the overall low death rate in our cohort. In the Italian outbreak in February 2020, while hospitalized patients had high numbers of comorbidities and were older age, severity of respiratory failure and renal impairment at presentation were predictors of 28-day mortality [10]. We surmise that predictors of hospitalization in a cohort of patients with mild illness may be different than the predictors of mortality in admitted patients with severe illness. 

Although limited in our ability to investigate predictive power of lymphopenia, partitioning analyses suggest that low lymphocyte counts associate with poor outcomes. Lymphopenia has been associated with adverse outcome in hospitalized patients with COVID-19 [11,12,13]. Here we show that even in patients presenting to the ambulatory setting with apparent mild illness, presence of lymphopenia at this early time point portends a poor prognosis with higher likelihood for subsequent complications and hospitalization. While patients presenting with apparent mild disease to the ambulatory setting do not typically undergo lab work (aside from the nasopharyngeal swab), obtaining a blood count may be a useful adjunct when there is doubt on the optimal triage for individual patients.

The role of SARS CoV-2 viral load in disease outcome remains unclear with conflicting reports [14]. In some studies, it has been associated with worse outcomes [15,16,17] while in others it has been reported to have no relationship with illness severity [18]. In our study, viral load did not correlate with disease severity or need for subsequent hospitalization. We also did not observe a significant difference in viral load between patients presenting to the ambulatory setting and an inpatient control. Thus, our findings do not support a significant effect of viral load in driving outcome, and we question the validity of using changes in viral load as a primary outcome in clinical trials. Furthermore, all viral load analyses need careful and cautious interpretation since factors such as specimen collection can impact Ct-values and the lack of a standard curve prevents calculation of the exact viral load. 

Much has been said about silent or "happy" hypoxemia in patients with COVID-19 that present with hypoxia and critical illness but without dyspnea [19,20]. By contrast, there has been little information on the predictive value of subjective SOB in patients with otherwise mild appearing illness and normal measurements of O_2_ saturations. In our cohort, SOB at initial visit was a strong predictor of subsequent clinical worsening and hospitalization. We observed SOB in 38.0% of the patients in our Combined Home Recovery group, similar to the 38% reported in a convenience sample of 81 adult patients treated on an outpatient basis from 16 participating states [21]. By contrast, 92.9% of the patients in our Subsequently Hospitalized group had SOB at initial presentation. Thus, presence of SOB at initial presentation is clearly a symptom that must alert both the patient and provider of the need for close monitoring if discharging to home, such as the use of a home O_2_ monitor and promptly returning to the hospital for worsening. 

Lungs from patients with acute respiratory distress syndrome due to COVID-19 show distinctive vascular features, consisting of severe endothelial injury with widespread thrombosis with microangiopathy [22]. Correlations exist between severe elevation of D-dimer levels and increase in the rate of complications among patients with COVID-19. The appropriateness of early D-dimer monitoring among patients with mild diseases deserves further investigation [23].

This study is not without limitations. Our cohort was from early in the pandemic when the full spectrum of symptoms in mild illness had yet to be defined; therefore, the prevalence of fever, cough, and SOB may have been overestimated while newer symptoms such as changes in smell and taste may not have been captured. There was also a lack of a standardized questionnaire for the initial patient encounter so some symptoms may not have been captured due to variations in provider approach to review of symptoms. In addition, we did not review convalescence during the period beyond the acute illness and recovery as this was outside the scope of this work. Finally, the number of patients in the Subsequently Hospitalized group was small (*n* = 14). This is good news in that 92% of the patients who were discharged home in the ambulatory setting went on to complete recovery at home. However, 14 patients required hospitalization, including 2 who died of respiratory failure. 

## 5. Conclusions

Presence of comorbidities was the single most reliable predictor of adverse outcome. Patients with multiple comorbidities had the most severe adverse events, with 100% of those with more than three comorbidities requiring hospitalization. In addition to comorbidities, subjective SOB and lymphopenia portend poor prognosis. These three factors, in this order, are most important for efficient triage at initial ambulatory visit. Patients with these risk factors should receive close monitoring and consideration for preemptive treatment soon after presentation to prevent worsening illness. Likewise, older age and male sex portended high risk for subsequent hospitalization. Conversely, younger age, female sex, absence of comorbidities, lack of SOB, and normal lymphocyte counts, predicted a good prognosis and safe home recovery. Targeted counseling, close follow up, and consideration for hospitalization, may improve outcomes in high risk patient populations.

## Figures and Tables

**Table 1:** Clinical Characteristics and Demographics of Patient Population based on Outcome.

Characteristics	Home Recovery No Complications (*N* = 153)	Home Recovery with Complications (*N* = 13)	Subsequently Hospitalized (*N* = 14)	*p* Value
Age				0.05 ^1^
*N*	153	13	14	
Median	52.6	56.5	56	
Range	(18.2–81.8)	(43.5–76.6)	(41.0–83.5)	
Female, *N* (%)	77 (50.3%)	4 (30.8%)	3 (21.4%)	0.06 ^2^
Duration from Symptom Onset (days)				0.54 ^1^
*N*	147	13	14	
Median	5	4	4	
Range	(1.0–60.0)	(1.0–14.0)	(1.0–12.0)	
Comorbidity, *N* (%)	61 (39.9%)	10 (76.9%)	12 (85.7%)	0.0003 ^2^
Race, *N* (%)				0.62 ^2^
Am. Indian, AK Nat., Nat. Hawaiian	5 (3.3%)	0 (0.0%)	0 (0.0%)	
Asian	20 (13.1%)	3 (23.1%)	2 (14.3%)	
Black, African American	9 (5.9%)	0 (0.0%)	0 (0.0%)	
Hispanic	19 (12.4%)	1 (7.7%)	3 (21.4%)	
Other	25 (16.3%)	1 (7.7%)	0 (0.0%)	
White	75 (49.0%)	8 (61.5%)	9 (64.3%)	
BMI Category, *N* (%)				0.63 ^3^
Obese	48 (39.0%)	6 (50.0%)	7 (50.0%)	
Overweight	40 (32.5%)	5 (41.7%)	5 (35.7%)	
Normal	34 (27.6%)	1 (8.3%)	2 (14.3%)	
Underweight	1 (0.8%)	0 (0.0%)	0 (0.0%)	
Lymphocyte × 10^9^/L				0.01 ^1^
N	20	7	7	
Median	1.6	1.1	0.3	
Range	(0.6–2.4)	(0.7–3.5)	(0.3–1.9)	
Neutrophil × 10^9^/L				0.87 ^1^
*N*	20	7	7	
Median	3.5	3.9	6.2	
Range	(1.5–8.9)	(1.8–5.1)	(0.8–11.5)	
Cycle Threshold				0.46 ^1^
*N*	79	10	7	
Median	15.1	16.8	12.6	
Range	(3.2–31.2)	(11.5–28.5)	(4.6–24.0)	

^1^ Kruskal–Wallis; ^2^ Chi-square; ^3^ Two-sided Fisher’s Exact.

## Data Availability

The data presented in this study are available on request from the corresponding author. The data are not publicly available due to ethical restrictions.

## Supplementary Materials

The following are available online at https://www.mdpi.com/article/10.3390/jcm10081562/s1, Figure S1: Association of symptoms with duration of illness before presentation; Figure S2: Association of symptoms with virus cycle threshold. Table S1: Symptom distribution in patient population based on outcome.
