# Supplementary material for: Early Prognostic Indicators of Subsequent Hospitalization in Patients with Mild COVID-19
Source: J Clin Med. 2021 Apr 8;10(8):1562. doi: 10.3390/jcm10081562 (PMC8068070; doi:10.3390/jcm10081562)
Supplement: Supplementary file 1 [file jcm-10-01562-s001.pdf]

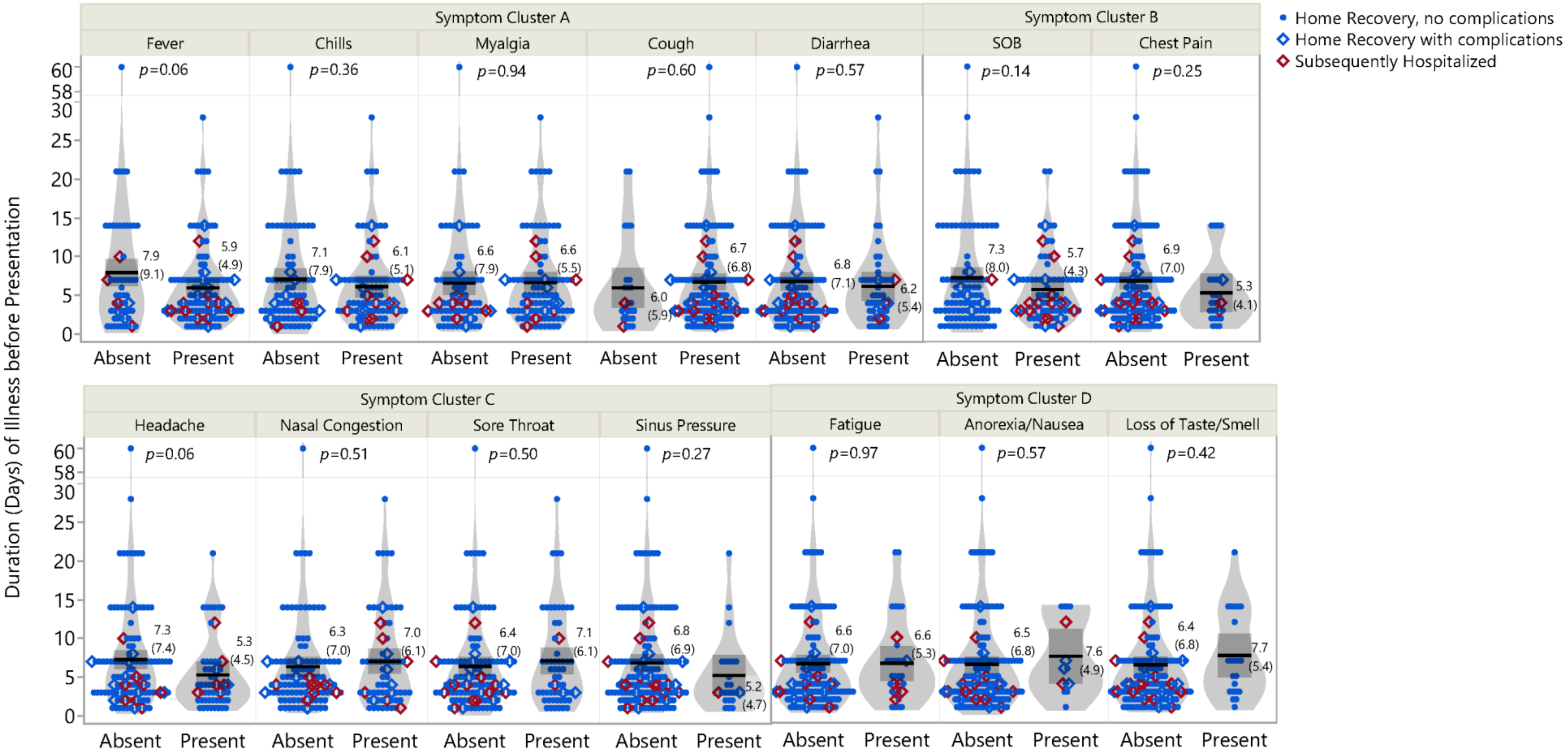

Figure S1. Association of symptoms with duration of illness before presentation. Duration of illness before presentation is shown based on the presence or absence of each of the 14 symptoms. Figures are annotated with mean (standard deviation), and *p*-value (*t*-test).

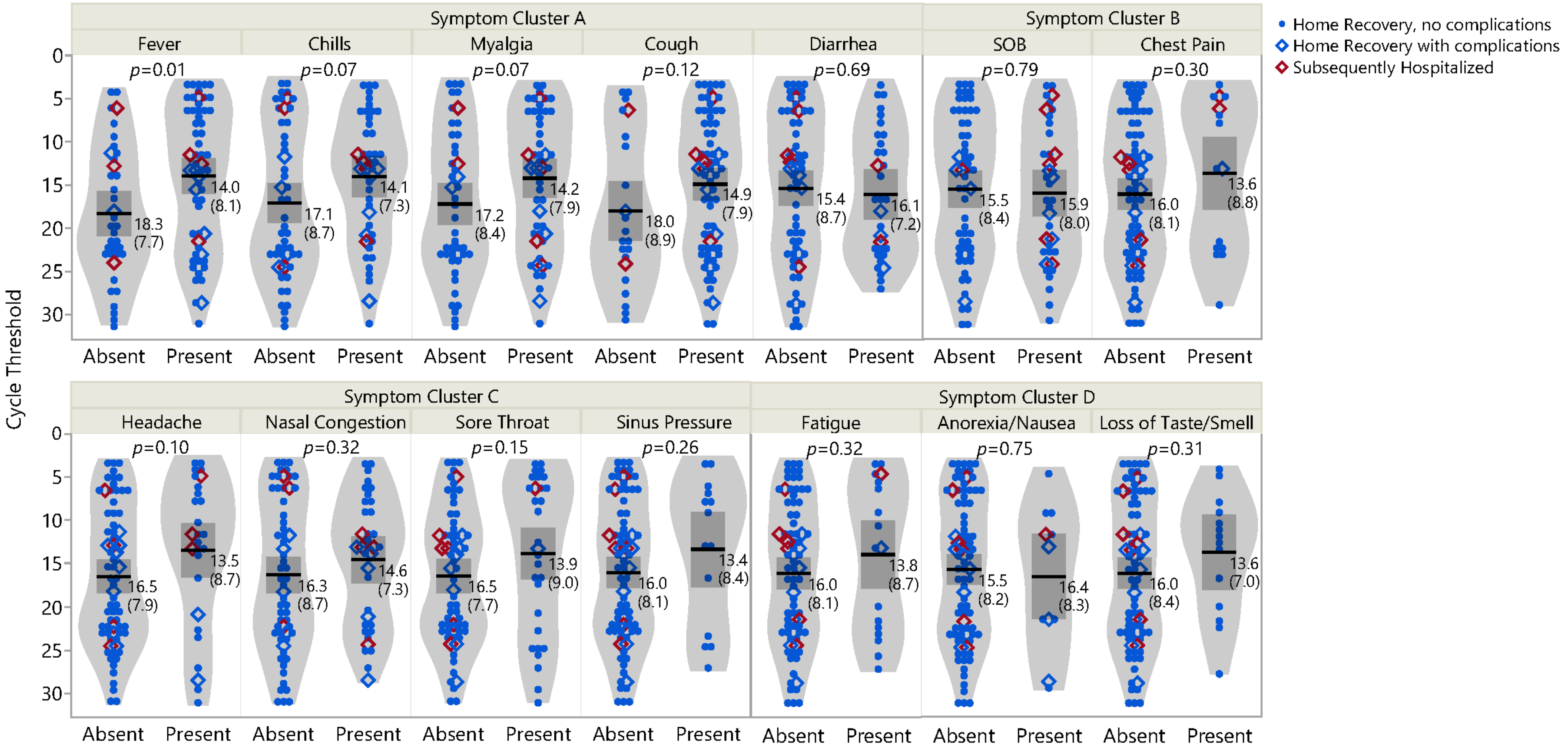

Figure S2. Association of symptoms with virus cycle threshold. Cycle threshold is shown based on the presence or absence of each of the 14 symptoms. Lower cycle threshold values indicate higher levels of viral nucleic acid. Figures are annotated with mean (standard deviation), and *p*-value (*t*-test).

Table S1. Symptom Distribution in Patient Population based on Outcome.

	Home Recovery No Complications (*N* = 153)	Home Recovery With Complications (*N* = 13)	Subsequently Hospitalized (*N* = 14)	*P* Value[1]
**Fever**				0.36
**Mild[2]**	58 (37.9%)	3 (23.1%)	6 (42.9%)	
**Moderate-Severe**	40 (26.1%)	7 (53.8%)	4 (28.6%)	
**Chills**	71 (46.4%)	7 (53.8%)	10 (71.4%)	0.19
**Myalgia**	82 (53.6%)	7 (53.8%)	7 (50.0%)	0.97
**Cough**	125 (81.7%)	12 (92.3%)	12 (85.7%)	0.77
**Diarrhea**	44 (28.8%)	4 (30.8%)	3 (21.4%)	0.89
**Headache**	49 (32.0%)	2 (15.4%)	6 (42.9%)	0.33
**Nasal Congestion**	55 (35.9%)	6 (46.2%)	6 (42.9%)	0.69
**Sore Throat**	54 (35.3%)	2 (15.4%)	2 (14.3%)	0.15
**Sinus Pressure**	22 (14.4%)	1 (7.7%)	1 (7.1%)	0.81
**SOB**	55 (35.9%)	8 (61.5%)	13 (92.9%)	**<0.0001**
**Chest Pain**	26 (17.0%)	1 (7.7%)	2 (14.3%)	0.91
**Fatigue**	27 (17.6%)	1 (7.7%)	6 (42.9%)	0.05
**Anorexia/Nausea**	10 (6.5%)	3 (23.1%)	2 (14.3%)	0.05
**Loss of Taste/Smell**	22 (14.4%)	0 (0.0%)	0 (0.0%)	0.17

[1]Chi-square or Two-Sided Fisher's Exact where symptom occurrence was low, [2]Temperature < 101° **F**
